# Étalonnage du test des cinq mots dans une population tunisienne de sujets sains

**DOI:** 10.11604/pamj.2019.34.58.14472

**Published:** 2019-09-30

**Authors:** Nadia Bouattour, Nouha Farhat, Hanen Hadjkacem, Olfa Hdiji, Salma Sakka, Mariem Dammak, Chokri Mhiri

**Affiliations:** 1 Service de Neurologie, Centre Hospitalo-Universitaire Habib Bourguiba, Sfax, Tunisie

**Keywords:** Test des 5 mots, mémoire, maladie d´Alzheimer, Five-word test, memory, Alzheimer’s disease

## Abstract

**Introduction:**

Le test des 5 mots (T5M) est un test de mémoire permettant d’explorer la mémoire verbale épisodique. Il évalue la mémoire des sujets ayant une plainte mnésique, en particulier dans le cadre du diagnostic de la maladie d’Alzheimer où il se révèle sensible et spécifique. L’objectif de notre travail est d’étudier l’effet des différents paramètres sociodémographiques sur les performances, d’établir des normes adaptées à la population tunisienne et de comparer nos résultats aux études précédentes.

**Méthodes:**

Nous rapportons l’étalonnage du T5M chez 315 sujets normaux âgés de 40 à 90 ans (169 hommes, 146 femmes), répartis en quatre tranches d’âges (40-49, 50-59, 60-69 et ≥ 70 ans) et trois niveaux éducatifs (I: primaire, II: secondaire et III: supérieur). Nous avons calculé la moyenne avec écart type pour les différents scores: Score Total (ST), Score Total Pondéré (STP), Rappel Différé Libre (RDL), Total Rappels Différés (TRD) et le Total Rappels Libres (TRL).

**Résultats:**

L’âge moyen était de 57.29 ans (±11.02). Nous avons constaté que les performances étaient meilleures chez sujets les plus jeunes et les mieux éduqués, sans différence significative entre les deux sexes. Les normes étaient calculées en fonction de l’âge et du niveau éducatif.

**Conclusion:**

Le T5M permet un dépistage rapide des patients chez qui une évaluation neuropsychologique complémentaire est souhaitable pour poser le diagnostic d’un trouble cognitif.

## Abstract (en)

**Introduction:**

Five-word test (5WT) is a memory test to assess the verbal episodic memory. It measures the memory of subjects with memory impairment, in particular within the framework of the diagnosis of Alzheimer’s disease, where it is sensitive and specific. The purpose of our study was to evaluate the effect of different sociodemographic parameters on subject’s performance, to set standards relevant to Tunisian population and to compare our results to previous studies.

**Methods:**

We report 5WT calibration in 315 normal subjects aged 40 to 90 years (169 men, 146 women), divided into four age groups (40-49,50-59,60-69 and 70 years) having three levels of education (I: primary, II:secondary and III:higher). We calculated the mean score (standard deviation) for the different scores: Total Score (TS), Total Weighted Score (TWS), Delayed Free Recall (DFR), Total Delayed Recall (TDR) and Total Free Recall (TFR).

**Results:**

The average age of subjects was 57.29 years (11.02). Performances appeared to be better in youngest and better educated subjects, without any significant difference between the two sexes. Standards were calculated on the basis of age and levels of education.

**Conclusion:**

Five-word test allows rapid screening of patients in whom complementary neuropsychological assessment is essential for the diagnosis of cognitive disorders.

## Introduction

La maladie d’Alzheimer (MA) est l’étiologie la plus fréquente parmi les syndromes démentiels, représentant 80% des cas de démence [1]. Des dégénérescences neurofibrillaires sont observées dès les premiers stades de la maladie [2] dans les structures temporales internes dont le rôle est crucial dans la mémoire épisodique [3]. L’évaluation des troubles de la mémoire constitue un élément essentiel dans le bilan neuropsychologique à visée diagnostique dans la MA. Les déficits mnésiques apparaissant à un stade très précoce et étant considérés comme un élément central dans le tableau des déficits cognitifs. Il est donc indispensable de disposer de tests évaluant spécifiquement la mémoire verbale qui constitue le domaine cognitif atteint en premier dans la MA. Ces tests doivent répondre à un nombre de critères: être rapidement administrée avec une durée de passation entre 5 et 10 minutes, avoir une bonne sensibilité pour détecter le maximum de personnes atteints de la MA et une bonne spécificité permettant de distinguer les sujets sains des personnes malades et être reproductibles d’un examinateur à l’autre. Il doit permettre aussi d’identifier en particulier le profil hippocampique ou non dans les troubles de la mémoire et de mieux guider le diagnostic différentiel essentiellement avec les difficultés de mémoire liées au vieillissement naturel [4-6]. La mémoire épisodique est habituellement évaluée à l’aide de tâches consistant à mémoriser une liste de mots, puis à restituer ces mots à l’aide d’un rappel libre. Plusieurs tests explorant ce domaine étaient rapportés dans la littérature on note: le test 5 mots, le test 16 mots de Grober et Buschke, le Selective Reminding Test (SRT)…

Le test des 5 mots (T5M) de Bruno Dubois: évalue rapidement la mémoire verbale sérielle en mesurant l’apprentissage et le rappel de cinq mots non prototypiques appartenant à des catégories sémantiques différentes. Il permet un apprentissage d’une liste de cinq mots au moyen d’un indiçage catégoriel initial. Ces indices catégoriels seront ultérieurement utilisés en cas de rappel libre déficient. Si la récupération des mots au moyen des indices est possible, on en déduit que les capacités de mémorisation étaient préservées mais qu’il existait une difficulté d’activation spontanée des stratégies de rappel: cette situation est classiquement observée chez les sujets normaux, les anxieux et les dépressifs ainsi que chez les patients atteints de dysfonctionnements sous-cortico-frontaux. À l’inverse, si le rappel reste déficient malgré l’indiçage, on peut conclure que le processus de mémorisation était structurellement insuffisant comme on le voit lors d’un déficit hippocampique [7].

Selon Dubois *et al.* [7], un score total inférieur à 10 à une sensibilité de diagnostic d’une MA de 91% et une spécificité de 87%. Pour le même score, Cowppli-Bony *et al.* [8] ont récemment montré une moindre sensibilité à 63% et une meilleure spécificité à 91%. Ces auteurs ont suggéré d’améliorer la performance de détection par une pondération qui double le poids des rappels libres par rapport aux rappels indicés. Ce score total pondéré varie de 0 à 20: pour un seuil de 17, la sensibilité de détection d’une MA est de 75,3% et la spécificité de 92,8%. L’évaluation de la mémoire verbale sérielle épisodique joue un rôle essentiel dans le diagnostic des démences. C’est pourquoi l’interprétation des résultats du test 5 mots nécessite des normes fiables. En l’absence des normes diagnostiques qui permettent de distinguer de façon optimale une population saine d’une population pathologique, le clinicien utilise souvent des normes comparatives permettant de situer les performances d’un individu par rapport à celles d’un échantillon de référence [9]. Cependant, en revoyant la littérature, nous avons constaté que l’étalonnage du test 5 mots était fait dans la population francophone ayant des caractéristiques sociodémographiques et linguistiques différentes de la population tunisienne et aucun étalonnage tunisien à ce jour n’a été publié, d’où l’intérêt de notre étude d’établir des normes pour le T5M permettant une meilleure évaluation de la population tunisienne.

Ainsi à travers cette première étude tunisienne nous nous proposons: d’étudier l’effet du sexe, de l’âge et du niveau éducatif de la population tunisienne sur les normes du T5M, d’établir des normes spécifiques à la population, de comparer nos résultats aux études précédentes, et de fournir un manuel pour les cliniciens adapté aux caractéristiques linguistiques et sociodémographiques de notre population permettant une meilleure évaluation cognitive.

## Méthodes

### Description de la population

Il s’agit d’une étude transversale sur une période de 12 mois (de juin 2015 jusqu’au juin 2016), dans laquelle l’inclusion des participants était prospective. Nous avons recruté sur la base du volontariat, des sujets âgés de 40 à 90 ans parmi les consultants, les visiteurs ou les accompagnants des malades, le personnel médical, quelques associations culturelles et chez des familles d’étudiants. Le recrutement des volontaires s’est effectué dans un bureau calme, loin de tout stimulus auditif ou visuel, par le même examinateur. Nous avons inclus dans cette étude des participants qui ont un âge compris entre 40 et 90 ans, ayant une autonomie pour les activités de la vie quotidienne basée sur une évaluation par la version abrégée de l’échelle « Instrumental Activites of Daily Living » (IADL) qui permet de préciser le degré du retentissement des troubles cognitifs sur la vie quotidienne. Le score total varie de 78 (en cas préservation de l’autonomie dans toutes les activités évaluées) à 0 (en cas de dépendance totale pour toutes les activités) [10]. Pour être inclus les sujets devraient avoir un Mini-Mental State Examination (MMSE) supérieur à 24/30. Les niveaux éducatifs étaient notés de I (le plus faible) à III (le plus élevé). Le niveau éducatif I correspondait aux sujets lettrés qui n’ont pas terminé leurs études primaires (MMSE > 24), le niveau éducatif II correspondait aux sujets qui ont terminé leurs études primaires et entamé les études secondaires mais n’ont pas eu leurs diplômes de baccalauréat (MMSE > 26) et le niveau III correspondait aux sujets ayant dépassé le baccalauréat (MMSE > 27) [11]. Nous avons éliminé de cette étude tous les sujets atteints de troubles graves de la perception visuelle ou auditive empêchant la réalisation des tests, les sujets atteints de maladie neurologique ou psychiatrique évolutive ou suspectée en particulier par la recherche d’un éventuel syndrome dépressif ou anxieux en utilisant l’échelle HADS « Hospital Anxiety and Depression Scale ». Un score supérieur à 7 constitue un critère d’exclusion [12]. Les sujets sous traitement neuroleptique ou psychotrope ou atteints d’une maladie générale grave pouvant perturber les fonctions cognitives et attentionnelles étaient exclus.

### Description de la tâche

Le T5M de Bruno Dubois [13] évalue rapidement la mémoire verbale sérielle en mesurant l’apprentissage et le rappel de cinq mots non prototypiques appartenant à des catégories sémantiques différentes. Le T5M comprend deux parties séparées d’une tâche cognitive intercurrente.

La première partie correspond à l’apprentissage de la liste des mots. Elle est réalisée en trois étapes successives que sont ***la présentation d’une liste de 5 mots*** en arabe (Musé, Limonade, Sautrelle, Camion, Passoire), ***l’encodage sémantique*** et ***le rappel immédiat*** (libre et indicé) qui vise à contrôler que les mots ont bien été enregistrés. 1) ***Présentation des mots*** disposés verticalement sur une feuille sont présentés au sujet, en lui demandant de s’en souvenir de façon explicite: « lisez cette liste de mots à voix haute et essayer de les retenir car je vous les redemanderai tout à l’heure ». 2) Puis l’on procède à ***« l’encodage sémantique »*** des mots en demandant au sujet: « pouvez-vous, tout en regardant la feuille, me dire quel est le nom de: l’ustensile de cuisine, du moyen de transport, de la boisson, du bâtiment, de l’insecte… ». 3) ***Le contrôle de l’enregistrement*** se fait par le rappel libre immédiat. Pour les mots qui ne sont pas rappelés en rappel libre, un rappel indicé est effectué et ne porte que sur les mots non rappelés : « quel était le nom de… ? ». Immédiatement après cette première partie, on effectue une ***tâche cognitive intercurrente*** qui est le ***test du cadran de l’horloge dans notre travail***.

La deuxième partie du test qui est l’étude de la mémorisation proprement dite par le rappel différé des cinq mots. Ce rappel est d’abord libre puis indicé pour les mots non rappelés spontanément selon la même procédure que lors du rappel immédiat.

#### Cotation et différents scores

Pour notre analyse, différents sous-scores ont été considérés. Les différents scores sont représentés dans le Tableau 1. Nous avons défini six variables quantitatives variant de 0 à 5: le Rappel libre immédiat (RLI), le Rappel indicé immédiat (RII), le Rappel libre différé (RLD), le Rappel indicé différé (RID), le Rappel immédiat ou score d’apprentissage (SA) = RLI + RII et le rappel différé ou Score de mémoire (SM) = RLD + RID. Deux variables quantitatives variant de 0 à 10, le score total (RI + RD) et le total rappel libre (RLI + RLD). Nous avons également mesuré deux pourcentages suggérés par Croisile B *et al.* [14]:

**Tableau 1:** Effectif et répartition en fonction de l’âge et du sexe et du niveau éducatif de l’échantillon d’étude

	Homme				Femme				Total (%)
Age (ans)	40-49	50-59	60-69	>70	40-49	50-59	60-69	>70	
Niveau éducatif I	18	15	17	25	24	33	26	8	166 (52,7)
Niveau éducatif II	12	13	21	8	10	14	9	0	87 (27,62)
Niveau éducatif III	9	9	12	10	10	5	7	0	62 (19,68)
Total (%)	39 (12,38)	37 (11,74)	50 (15,87)	43 (13,65)	44 (13,97)	52 (16,5)	42 (13,33)	8 (2,25)	315 (100)

**L’indiçage immédiat** = [RII×100] / [5 - RIL] et **l’indiçage différé** = [RDI×100] / [5 - RDL]

Ces deux pourcentages sont plus intéressants que la note brute du rappel immédiat indicé ou du rappel différé indicé. Ils permettent d’apprécier la capacité à récupérer, grâce à la facilitation de l’indice catégoriel, l’information certes stockée mais dont le rappel spontané s’est révélé impossible. Nous avons calculé aussi le score total des rappels libres (TRL), correspondant à la somme du rappel immédiat libre et du rappel différé libre (TRL = RIL + RDL). Ce score évalue la facilité de réalisation des rappels libres c’est-à-dire la capacité spontanée de réponse. Nous avons calculé le taux d’oubli qui est la différence entre le score d’apprentissage et le score de mémoire selon la formule suivante:

**Taux d’oubli = [100× (SA - SM)] / SA**

Cette mesure permet d’appréhender ***le déficit de stockage de l’information en mémoire***. Nous avons calculé aussi un score suggéré par Cowppli-bony *et al.* [8] le Score Total pondéré (STP) qui majore le poids des réponses libres selon la formule suivante: **STP = 2RLI + RII + 2RLD + RID**. Ce score varie de 0 à 20.

### Analyse statistique

La distribution de chaque score de test a été analysée pour les trois variables sociodémographiques suivantes: le sexe, le niveau éducatif et l’âge. Quatre tranches d’âge (40-49 ans, 50-59 ans, 60-69 ans et supérieur ou égal à 70 ans) et trois niveaux éducatifs ont été constitués. Les traitements statistiques ont été effectués avec le logiciel Microsoft Office Excel XL STAT 2014. Nous avons utilisé: des analyses de variance « ANOVA », en introduisant systématiquement trois facteurs inter sujets: l’âge (4 tranches), le niveau d’éducation (3 niveaux), le sexe (2 niveaux). Le test de « student » était utilisé pour la comparaison des 2 moyennes. Le test de « Chi 2 » était utilisé pour la comparaison des pourcentages. Les corrélations entre les résultats des différents tests étaient réalisées avec des tests non paramétriques. Le seuil de signification (p) était fixé à 0,05. Par ailleurs, pour la présentation des normes, nous avons calculé les moyennes avec les écarts type et les seuils de normalité.

## Résultats

Le groupe des volontaires est formé de 315 sujets, l’âge moyen était de 57,29 ans (±11,02). Parmi nos participants 53,65% (169) étaient des hommes et 46,35 % (146) étaient des femmes. Le Tableau 1 représente la répartition de la population en fonction de l’âge, du sexe et du niveau éducatif. Le MMSE moyen était de 27.51 (±2.22).

### Influence des paramètres sociodémographiques

***Influence du sexe:*** la comparaison entre hommes et femmes des moyennes globales des différents scores du T5M en utilisant le test de « Student » n’a pas montré de différence significative. Cette différence reste non significative pour tous les scores en comparant les 2 sexes du même niveau éducatif et de la même tranche d’âge.

***Influence de l’âge:*** la comparaison des moyennes des différents scores du T5M en fonction de l’âge en utilisant le test d’ANOVA a montré que l’effet de l’âge était variable selon le score. Ainsi les scores TRL (P = 0.02) et STP (P = 0.02) étaient significativement liés à l’âge, contrairement aux scores RIL, RII, SA, RDL, RID, SM et ST ou la différence observée n’était pas significative (Tableau 2).

**Tableau 2:** Variabilité des normes du test de 5 mots en fonction de l’âge et du niveau éducatif

AGE (ans)	RIL/5	SA/5	RDL/5	SM/5	TRL/10	Taux d’oubli	ST/10	STP/20
40-49 (N= 83)	4,66	4,93	4,67	4,95	9,34	-0,73	9,87	19,22
50-59 (N= 89)	4,53	4,91	4,54	4,85	9,07	0,65	9,80	18,84
60-69 (N= 92)	4,4	4,84	4,53	4,89	8,93	-1,67	9,71	18,65
70 (N= 51)	4,39	4,84	4,39	4,9	8,78	-1,55	9,74	18,53
P-Value	0,055	0,2	0,13	0,29	**0,02**	0,47	0,15	**0,02**
Niveau éducatif	**RIL/5**	**SA/5**	**RDL/5**	**SM/5**	**TRL/10**	**Taux d’oubli**	**ST/10**	**STP/20**
Niveau éducatif I (N= 166)	4,35	4,38	4,42	4,85	8,78	-1,26	9,68	18,45
Niveau éducatif II (N= 87)	4,57	4,92	4,61	4,91	9,18	-0,43	9,85	19,03
Niveau éducatif III (N= 62)	4,8	4,97	4,81	5	9,61	-0,79	9,95	19,58
P-Value	**<0,05**	**0,01**	**<0.05**	**0,01**	**< 0,05**	0,83	**< 0,05**	**< 0,05**

RIL: Rappel Immédiat Libre, SA : Score d’Apprentissage, RDL : Rappel Différé Libre, SM : Score de Mémoire, ST : Score Total, STP : Score Total Pondéré

***Influence du niveau éducatif:*** l’analyse des variances en fonction du niveau éducatif a montré que tous les scores du T5M sont significativement liés au niveau éducatif, sauf le taux d’oubli qui n’était pas influencé (Tableau 2).

### Normes des scores du test 5 mots en fonction de l’âge et du niveau éducatif

Dans ce chapitre nous avons procédé à une analyse des différents scores du T5M en tenant compte à la fois de l’âge et du niveau scolaire, ce qui nous a permis de distinguer 12 sous-groupes. Le choix de ces 2 paramètres sociodémographiques pour établir des normes repose sur l’influence de ces 2 facteurs sur les performances des différents scores du T5M, contrairement au sexe qui n’était pas un facteur déterminant. Nous avons étalonné le T5M chez 315 sujets normaux âgés de 40 à 90 ans. Pour chaque tranche d’âge et chaque niveau éducatif, le médecin pourra établir ses cut -off à - 1,5 ou - 2 déviation Standard. Le Tableau 3 indique les performances des quatre tranches d’âge en fonction des trois niveaux éducatifs pour les scores du T5M. Les scores de RII et de RID ne sont pas indiqués car ces scores bruts sont moins intéressants que les pourcentages d’indiçage immédiat et différé.

**Tableau 3:** Normes du test 5 de mots en fonction de l’âge et du niveau scolaire

Scores/âge (ans)	Niveau scolaire I				Niveau scolaire II				Niveau scolaire III			
	40-49	50-59	60-69	≥ 70	40-49	50-59	60-69	≥ 70	40-49	50-59	60-69	≥ 70
**RIL/5**	4,52 (±0,7)	4,45 (±0,68)	4,2 (±0,7)	3,5 (±0,84)	4,77 (±0,43)	4,6 (±0,64)	4,37 (±0,85)	4,75 (±0,46)	4,84 (±0,37)	4,64 (±0,84)	4,89 (±0,31)	5
**SA /5**	4,9 (±0,3)	4,87 (±0,33)	4,74 (±0,44)	4,79 (±0,41)	4,95 (±0,21)	4,92 (±0,38)	4,9 (±0,3)	4,87 (±0,35)	4,95 (±0,23)	5	4,95 (±0,23)	5
**RDL/5**	4,55 (±0,74)	4,48 (±0,74)	4,37 (±0,76)	4,24 (±0,79)	4,86 (±0,39)	4,48 (±0,64)	4,63 (±0,55)	4,25 (±0,7)	4,74 (±0,56)	4,86 (±0,53)	4,74 (±0,56)	5
**SM /5**	4,93 (±0,34)	4,8 (±0,39)	4,84 (±0,37)	4,84 (±0,36)	4,95 (±0,21)	4,85 (±0,46)	4,9 (±0,4)	5 (±0,0)	5	5	5	5
**TRL/10**	9,07 (±1,18)	8,94 (±1,06)	8,58 (±1,00)	8,42 (±1,3)	9,64 (±0,66)	9,07 (±1,03)	9 (±1,17)	9 (±0,92)	9.56 (±0,7)	9,5 (±1,09)	9,63 (±0,83)	9,8 (±0,42)
**Taux d’oubli**	- 0,95 (±10,43)	-1,87 (±24,42)	-2,9 (±13,15)	-1,97 (±11,45)	-0,22 (±6,98)	3,7 (±11,14)	-1 (±22,74)	-11,46 (±23,96)	-1,32 (±5,73)	0	-1,32 (±5,73)	0
**ST/10**	9,83 (±0,44)	9,7 (±0,5)	9,53 (±0,63)	9,64 (±0,6)	9,9 (±0,29)	9,85 (±0,46)	9,8 (±0,55)	9,87 (±0,35)	9,89 (±0,31)	10	9,95 (±0,23)	10
**STP/20**	18,9 (±1,41)	18,6 (±1,52)	18,14 (±1,44)	18,06 (±1,7)	19,54 (±0,86)	18,92 (±1,3)	18,8 (±1,52)	18,87 (±1,12)	19,53 (±0,84)	19,5 (±1,09)	19,58 (±1,02)	19,8 (±0,42)

RIL : Rappel Immédiat Libre, SA : Score d’Apprentissage, RDL : Rappel Différé Libre, TRL : Total des rappels libres, SM : Score de Mémoire, ST : Score Total, STP : Score Total Pondéré

La survenue de score de zéro est rare. Aucun des 315 sujets n’a obtenu de note de zéro aux six scores suivants: ST, STP, RIL, SA, SM et TRL. La note maximale de 10 a été obtenue au ST par 256 sujets (81,30%) répartis de la manière suivante (28,51%) des sujets de la tranche d’âge de 40-49 ans, ( 28,9 %) des sujets de la tranche d’âge de 50-59 ans, (27,34 %) des sujets de la tranche d’âge de 60-69 ans et (15,62%) des sujets de la tranche d’âge ≥ 70 ans. Les autres notes obtenues au ST: 9 chez 48 sujets (15.24 %), 8 chez 10 sujets (3,17%). Aucun participant n’avait donc de Score Total ≤ 7. Un STP égale à 20 était obtenu par 144 sujets (45,71%), une note comprise entre 20 et 17 était obtenu par 142 sujets (45,07 %), la note 17 étant le seuil minimal pour le STP défini par Cowppli-Bony *et al.* [8].

### Le taux d’oubli

Le taux d’oubli n’est pas significativement influencé par l’âge (p = 0,464), ni par le sexe (p = 0,45) et le niveau éducatif (p = 0,825) selon l’analyse des variances.

***L’indiçage:*** l’intérêt du T5M est de permettre un apprentissage indicé des cinq mots, indiçage utilisé ensuite pour faciliter le rappel immédiat ou différé des mots non rappelés spontanément.

***Indiçage immédiat:*** lors du rappel immédiat, 192 sujets sur 315 ont obtenu le score maximum de 5 au RIL (60,95%). Ainsi, 123 sujets auront besoin d’un indiçage car leurs scores de RIL était de 4 chez 95 sujets, 3 chez 23 sujets et 2 chez 5 sujets. Les sujets qui ont nécessité l’indiçage se répartissent en: 84 sujets de niveau éducatif I (50.6% des 166 sujets), 29 sujets de niveau éducatif II (33,33 % des 87 sujets), et 10 sujets de niveau éducatif III (16,13 % des 62sujets). Selon l’âge, il s’agissait de 24 sujets de la première tranche d’âge (28,91% des 83 sujets), 33 sujets de la deuxième tranche d’âge (37,07% des 89 sujets), 42 sujets de la troisième tranche d’âge (45,65 % des 92 sujets), et 24 sujets de la quatrième tranche d’âge (47,05 % des 51 sujets). Nous avons constaté donc que l’indiçage est plus souvent nécessaire chez les sujets les plus âgés (47,05% des sujets de plus de 70 ans) et les moins éduqués (50,6% des sujets du niveau éducatif I).

***Indiçage différé:*** lors du rappel différé, 202 sujets sur 315 ont obtenu un score maximal au RDL (64,13%). Ainsi, comparativement à l’indiçage immédiat nécessaire chez 123 sujets (39,04 %), l’indiçage différé était nécessaire chez un nombre moins élevé de sujets: 112 sur 315 (35,55%) sans différence significative (p = 0,38 selon le test de Chi-deux). Un indiçage différé était nécessaire chez 73 sujets de niveau éducatif I (43,97%), 30 sujets de niveau éducatif II (34,48%) et 9 sujets de niveau éducatif III (14,51%). Selon l’âge, il s’agissait de 21 sujets de la première tranche d’âge (25,3%), 32 sujets de la deuxième tranche d’âge (35,95%), 35 sujets de la troisième tranche d’âge (38,04%), et 24 sujets de la quatrième tranche d’âge (47,05%). Nous avons constaté là-aussi que l’indiçage est plus souvent nécessaire chez les sujets les plus âgés (47,05% des sujets de plus de 70 ans) et les moins éduqués (43,97% des sujets de niveau éducatif I).

***Efficacité de l’indiçage:*** lors du rappel immédiat, 123 sujets avaient eu besoin d’un indiçage: 101 d’entre eux avaient un indiçage efficace à 100% (82,11%) alors que 22 sujets ont raté leurs RII (17,89%). Au total, 293 des 315 sujets testés (93,01%) avaient donc un TRI (SA) maximal égal à 5. En revanche, l’indiçage n’était pas aussi efficace lors du rappel différé: sur les 112 sujets qui avaient eu besoin d’un indiçage, seulement 90 (73,77%) ont réussi à normaliser complètement leur rappel différé. Au total, 292 sujets sur 315 (92,70%) avaient un TRD (SM) maximal égal à 5. Ainsi, sur l’ensemble des 315 sujets, seulement 144 (45,71%) avaient d’emblée un score total égal à 10 (égal au TRL), et avec l’indiçage, 256 sujets (81,27 %) qui avaient donc un score total égal à 10. Si nous comparons ces 256 sujets ayant eu un Score Total égal à 10 aux 59 autres qui avaient un score total de 8 ou 9, nous constatons qu’ils étaient plus jeunes (56,43 ans (±11,54) versus 60,33 ans (±10,53); p= 0,013).

## Discussion

Le T5M est un test de mémoire épisodique verbale conçu pour détecter un trouble de mémoire chez des sujets âgés développant une maladie d’Alzheimer [13, 15]. Sa réalisation s’assure d’un bon encodage lors de l’apprentissage. Le manque de rappel spontané immédiat ou différé est facilité par un indiçage catégoriel. À ce jour, seul un seuil pathologique a été défini. Face au succès indéniable du T5M [16], il est important de disposer des normes, en particulier pour les tranches d’âges élevés et les bas niveaux éducatifs.

### Le Score total

Un score total inférieur à la note maximale de 10 entraîne une sensibilité de diagnostic d’une MA de 91% et une spécificité de 87% selon l’étude de Dubois *et al.* [13], ou bien une sensibilité à 63% et une spécificité à 91% selon l’étude de Cowppli-Bony *et al.* [8]. C’est ainsi qu’un score total inférieur à la note maximale de 10 amène légitimement à se poser la question d’un processus pathologique. Notre étude a montré que sur l’ensemble de nos 315 sujets normaux, seulement 140 sujets (44.44%) avaient d’emblée un score égal à 10 (équivalent au TRL), et avec l’indiçage, ce sont 256 sujets (81,26 %) qui avaient un score total égal à 10. Nos résultats sont conformes à l’étude de Croisile *et al.* [14].

### Le temps d’interférence entre rappel immédiat et rappel différé

Par rapport au T5M préconisé par croisile *et al.* [14], notre travail comporte une différence dans le temps d’interférence entre les rappels immédiats et différé, 5 minutes dans notre étude contre 20 minutes dans l’étude de croisile *et al.* Ce temps était préconisé par Dubois *et al.* [13]. Nous insistons sur la nécessité d’une interférence d’au moins cinq minutes entre le rappel immédiat et le rappel différé. Cette interférence doit être impérativement consacrée à la réalisation d’autres tests cognitifs afin de sensibiliser une difficulté de consolidation et de stockage à long terme des mots mémorisés. Dans notre étude le test de l’horloge était utilisé comme interférence. Sans cette précaution d’une longue interférence cognitive, le T5M ne remplit pas pleinement son rôle de test de mémoire à long terme.

### L’indiçage

L’indiçage était aussi bien nécessaire lors du rappel différé que du rappel immédiat sans différence significative entre les 2 rappels. Concernant l’efficacité de l’indiçage, il a été démontré que les sujets normaux ayant des difficultés de rappel libre normalisaient en grande partie leurs performances lors du rappel indicé de ces mots non rappelés spontanément [5, 6, 17]. Notre étude confirme ce point en montrant une efficacité quasi-complète de l’indiçage chez 82,3 % des sujets ayant eu un RIL déficient, ce qui aboutit à un excellent TRI (SA) puisque 93.01% des sujets auront la note maximale de 5.

Parallèlement, l’indiçage normalise 73,77 % des sujets ayant eu un RDL déficient ce qui permet à 92,7% des sujets d’avoir un TRD (SM) maximal de 5. Ces résultats étaient légèrement différents par rapport à ceux observées dans l’étude de Croisile *et al.* ou l’indiçage était sept fois plus souvent nécessaire lors du RD que du RI, avec une meilleure efficacité pour le RI (efficacité complète de l’indiçage chez 93,8% des sujets ayant eu un RIL déficient (82,11% dans notre étude), ce qui aboutit à un excellent SA puisque 99,5% des sujets auront la note maximale de 5 (93,01% dans notre étude). En revanche, l’indiçage n’a normalisé que 55,1 % des sujets ayant eu un RDL déficient (73,77% dans notre étude) permettant à 74,9 % des sujets d’avoir un SM maximal de 5 (versus 92,7 % dans notre étude) [14].

Rôle des variables sociodémographiques

Notre étude montre que les performances du T5M sont sensibles aux effets de l’âge et du niveau éducatif mais non à ceux du sexe. La littérature neuropsychologique montre que la plupart des tests cognitifs sont sensibles au niveau éducatif [18]. Cette influence du niveau d’éducation sur les performances cognitives est double: d’une part, les sujets de faible niveau éducatif sont constitutionnellement moins efficaces que les sujets de niveau plus élevé, d’autre part, les sujets de faible niveau éducatif perçoivent souvent la situation de passation de tests comme scolaire et l’anxiété qui en résulte réduit leur implication lorsqu’ils subissent ces tests. Ce résultat démontré dans notre étude n’était pas observé dans l’étude de Croisile *et al.* [14], ou le niveau éducatif n’avait pas d’effet sur la performance des scores du T5M.

**Effet de l’âge: sujets âgés de plus de 70 ans:** l’effectif de notre groupe de sujets d’âge supérieur à 70 ans est le plus faible (51 sujets), mais non négligeable. Dans la littérature la majorité des études des tests de la mémoire incluent plutôt des sujets âgés plus de 80 ans avec des échantillons très faibles: l’étalonnage du RI-48 ne comporte que 12 sujets de 80 à 90 ans, et celui du DMS48 comporte 20 sujets de 80 à 90 ans et 7 de 90 à 100 ans [19].

Le choix de cut-off de 70 ans dans notre étude était lié d’une part aux particularités sociodémographiques de la population tunisienne dont l’espérance de vie se situe aux alentours de 72,5 ans pour les hommes et 76,5 ans pour les femmes, d’autre part au niveau éducatif faible de la majorité des octogénaires ce qui nous a empêché de trouver un échantillon représentatif et d’établir des normes pour cette catégorie d’âge. Dans notre étude, tous les scores du T5M obtenus pour la tranche des sujets de 70 ans (70 à 80 ans) sont inférieurs à ceux des trois autres tranches d’âge, mais la différence n’était significative que pour les scores TRL et STP.

La question que l’on peut donc se poser est la suivante: cette classe des 70 ans se distingue-t-elle des autres uniquement parce que leur âge avancé rend plus fragiles, de manière physiologique, leurs processus de mémorisation ou bien parce que ces sujets ne sont pas tous normaux ? Cette question est cruciale à chaque fois qu’il s’agit d’étalonner n’importe quel test cognitif. Nous rappelons que nos sujets étaient parfaitement autonomes et qu’ils ne remplissaient aucun des critères des principales démences dégénératives connues. Lors de l’étalonnage d’un test, on ne peut toutefois jamais exclure que les tranches d’âges élevés contiennent en leur sein quelques individus supposés normaux mais en fait au début d’un processus dégénératif. Les performances de ces sujets déjà déficitaires mais sans être déments contaminent les moyennes de cette classe d’âge, faussent la pertinence de l’échantillon et rendent ensuite plus difficile le repérage par ce test des sujets pathologiques de la population générale lorsque l’on utilisera ces normes.

### Un profil type des sujets normaux ?

À partir des résultats des sujets normaux, nous suggérons que la présence de certains éléments, lors de la passation du test du T5M, évoquerait fortement un processus pathologique, le clinicien est légitimement en droit de s’inquiéter lorsque se combinent les situations suivantes: indiçage immédiat inefficace, score total inférieur ou égal à 6, note de zéro à un des scores suivants: STP, RIL, TRI [SA], TRD [SM] et TRL [14]. Si un score total inférieur à 10 n’est pas forcément pathologique puisqu’il a été observé chez près de 18,76 % des sujets normaux dans notre étude, il incite néanmoins à la prudence et la nécessité de surveiller ces sujets sans forcément réaliser un examen neuropsychologique plus poussé.

## Conclusion

Notre étude confirme que le T5M permet d’explorer la mémoire verbale sérielle, qui est un test d’utilisation facile quel que soient l’âge et le niveau éducatif du sujet testé. Nous avons constaté que les performances de nos sujets normaux sont influencées uniquement par l’âge et le niveau éducatif, contrairement au sexe qui n’était pas un facteur influençant. Les performances étaient meilleures chez les sujets les plus jeunes et les mieux éduqués. Nos résultats étaient proches de la littérature en quelques points et différents dans d’autres. Au terme de cet étalonnage tunisien nous nous proposons des normes adaptées aux caractéristiques linguistiques et sociodémographiques de la population tunisienne, permettant une meilleure évaluation cognitive. Une limite importante de notre étude est due au fait que certaines tranches d’âge étaient peu représentées dans notre population de référence tel que l’insuffisance de l’effectif de la quatrième tranche d’âge (supérieur à 70 ans) appartenant aux niveaux éducatifs II et III. De ce fait les données normatives que nous avons proposées pour cette tranche d’âge devraient être utilisées avec prudence.Notre étude confirme que le T5M permet d’explorer la mémoire verbale sérielle, qui est un test d’utilisation facile quel que soient l’âge et le niveau éducatif du sujet testé. Nous avons constaté que les performances de nos sujets normaux sont influencées uniquement par l’âge et le niveau éducatif, contrairement au sexe qui n’était pas un facteur influençant. Les performances étaient meilleures chez les sujets les plus jeunes et les mieux éduqués. Nos résultats étaient proches de la littérature en quelques points et différents dans d’autres. Au terme de cet étalonnage tunisien nous nous proposons des normes adaptées aux caractéristiques linguistiques et sociodémographiques de la population tunisienne, permettant une meilleure évaluation cognitive. Une limite importante de notre étude est due au fait que certaines tranches d’âge étaient peu représentées dans notre population de référence tel que l’insuffisance de l’effectif de la quatrième tranche d’âge (supérieur à 70 ans) appartenant aux niveaux éducatifs II et III. De ce fait les données normatives que nous avons proposées pour cette tranche d’âge devraient être utilisées avec prudence.

### État des connaissances actuelles sur le sujet

- Des manifestations bucco-dentaires peuvent exister au cours de certaines maladies rares, et impacter négativement sur la santé bucco dentaires et au niveau fonctionnel;
- Des soins spécifiques sont souvent nécessaires chez ces parents;
- La question est insuffisamment étudiée en Afrique.

### Contribution de notre étude à la connaissance

- Évaluation pionnière du problème dans notre contexte;
- Cette étude nous apporte des éléments de plaidoyer pour l'amélioration objective de l'offre de soins à cette catégorie de patients souvent marginalisés.

## Conflits d’intérêts

Les auteurs ne déclarent aucun conflit d’intérêts.
